# Role of transcutaneous electrical nerve stimulation in alleviation of tinnitus in normal hearing subjects

**DOI:** 10.1007/s00405-024-09182-y

**Published:** 2025-01-17

**Authors:** Maha Abdelgaber A. Aly, Enas Mostafa O. Ramadan, Amira Mohammad Eloseily

**Affiliations:** Audio-vestibular Medicine unit, department of Ear, Nose and throat, Faculty of Medicine, Assiut University, Assiut, Egypt

**Keywords:** Subjective tinnitus, TENS, Visual analog scale, Transcutenous auricular vagus nerve stimulation

## Abstract

**Background:**

Subjective tinnitus is characterized by perception of sound in the absence of any external or internal acoustic stimuli. Many approaches have been developed over the years to treat tinnitus (medical and nonmedical). However, no consensus has been reached on the optimal therapeutic approach. Electrical nerve stimulation targeting peripheral auditory pathways presents a promising area of investigation for the treatment of tinnitus. Non-invasive transcutaneous vagal nerve stimulation of the auricular branch of the vagus nerve has been introduced and studied but its success rate varies and conflicting results have been reported. In this study we aim to assess the role of transcutaneous electrical nerve stimulation in alleviation of tinnitus in normal hearing subjects and to study the different factors that may affect the degree of alleviation of tinnitus.

**Methods:**

The study group consisted of 64 subjects (38 male and 26 female). The age ranged between 20 and 60 years. All of them suffering from subjective tinnitus. Assessment of tinnitus loudness analyzed by Visual Analogue Scale (VAS) which was applied before and directly after TENS stimulation.

**Results:**

There is a statistically significant difference in tinnitus loudness before and after TEN stimulation as assessed by VAS. 45 out of 64 (70.31%) patients had improvement after TENS, from them nine patients had a complete reduction of tinnitus. There is no statistically significant relation between the studied variables and the degree of tinnitus reduction.

**Conclusion:**

ta-VNS is an effective treatment of subjective tinnitus but we could not assess for how long this residual inhibition persists due to lack of long term follow up. However, it is difficult to decide who might benefit from ta-VNS, patients with unilateral tinnitus on right ear and those with whistling sound have more reduction in tinnitus loudness than others.

## Background

Tinnitus is a common complaint divided into two broad groups, objective and subjective tinnitus. Subjective tinnitus is characterized by perception of sound in the absence of any external or internal acoustic stimuli (phantom sensation). It can be perceived as a hissing, buzzing, humming, roaring, whistling, or ringing sound in one or both ears, or somewhere in the head. Tinnitus is a prevalent condition affecting 12–30% of the general population worldwide [1, 2]. Its prevalence does not differ by sex but increases with age, affecting 10% of young adults, 14% of middle‑aged people, and 24% of adults as a whole [3, 4]. and the prevalence is expected to continue rising, indicating a growing global burden [5–7].

The underlying pathophysiology of tinnitus is poorly understood, but auditory differentiation secondary to otologic injury is thought to give rise to abnormal firing of nerves within central auditory pathways, creating what patients experience as a phantom sensation [8]. Studies in tinnitus patients without any hearing abnormalities suggest that the non-auditory brain network also plays a specific role in the occurrence and development of tinnitus. These networks include attention, salience, distress, and memory function. The activation of these non-auditory networks also leads to comorbidities, such as sleep disorder, anxiety, and depression. In general, tinnitus occurs due to changes in the neuroplasticity of the auditory and non-auditory systems [9]. Tinnitus can have a profound impact on the social functioning and psychological well-being of patients. It can be associated with sleep disturbance, social withdrawal, anxiety, and depression [10–12].

Many approaches have been developed over the years to treat tinnitus. Common medical management of tinnitus mainly includes pharmacological treatment (e.g., vasodilators, antidepressants, anticonvulsants, anxiolytics or intratympanic medications) and non- pharmacological therapies including sound therapy, cognitive–behavioral therapy, transcranial magnetic stimulation, hearing aids, education and counselling [13]. However, no consensus has been reached on the optimal therapeutic approach [9, 14].

The use of electricity to suppress tinnitus has been tried as early as 1886 [15]. Electrical nerve stimulation targeting peripheral auditory pathways presents a promising area of investigation for the treatment of tinnitus. Transcutaneous electrical nerve stimulation (TENS) is a noninvasive, very safe method of delivering electrical current to a nerve through the skin [16]. If electrical stimulation is applied to the auricular branch of the vagus nerve (ABVN) it is terminologically called transcutaneous auricular VNS (ta-VNS) [17]. The ABVN is mainly distributed in the concha (cymba conchae and cavum conchae), and the cymba conchae is supplied exclusively by the ABVN [18]. To differing degrees, the ABVN also dominates other auricle regions. The antihelix, cavum conchae, tragus, crus of helix, and crura of anthelix are 73%, 45%, 45%, 20%, and 9% dominated by the ABVN, respectively [19].

When the different areas of the ABVN distribution are stimulated, there are varying degrees of activation along the VN pathway. The afferent fibers of the ABVN enter the vagal trunk through the jugular ganglion and project to the nucleus of the solitary tract [20], where the central integration of autonomic neurons occurs. It collects afferent information and activates the caudal ventrolateral medulla and dorsal motor nucleus to regulate central autonomic activity [21]. ta-VNS is being explored as a potential treatment option for tinnitus [22]. The mechanisms may involve modulation of multiple brain regions related to the emotional, auditory and attentional aspects of tinnitus [23, 24].

Non-invasive transcutaneous vagal nerve stimulation (tVNS) of the auricular branch of the vagus nerve has been introduced and studied [25–29] but its success rate varies and conflicting results have been reported [30]. In this study we aim to assess the role of transcutaneous electrical nerve stimulation in alleviation of tinnitus in normal hearing subjects and to study the different factors that may affect the degree of alleviation of tinnitus.

## Subjects and method

### Subjects

The study is a prospective cross-sectional study conducted in Audiovestibulr Medicine unit, Assiut University hospital, Assiut, between November 2023and August 2024. The study group consisted of 64 subjects of both sex, all of them suffering from subjective tinnitus.

Subjects with hearing loss, middle ear pathology, objective tinnitus, history of head trauma, neurological or psychiatric illness, cerebrovascular disease, electrolyte imbalance, a cardiac pacemaker, metal implants and pregnancy were excluded from this study.

### Ethical consideration

The proposal was reviewed and fulfilled all requirements as governed by Declaration of Helsinki. Ethical approval was obtained by the ethical committee, Faculty of Medicine, Assiut University, Egypt. IRB approval number 042023300292. Informed consent was obtained from all participants in the study.

### Methods

Detailed medical history was taken through personal interviewing of participants focusing on tinnitus duration, sound, laterality, associated symptoms with tinnitus, previous treatment and family history of tinnitus, history of noise exposure as well as history of chronic disease.

All subjects undergo a basic audiological evaluation using a dual channel clinical audiometer (Orbiter 922, GN Otometrics, Cobenhagen, Denmark) to assess pure tone and speech audiometery, and immittancemetry measurement using (Impedance Audiometer Interacoustic AT 235, Denmark) to assess middle ear function.

Assessment of tinnitus loudness analyzed by Visual Analogue Scale (VAS) which was applied before and directly after TENS. A Visual Analogue Scale (VAS) is a measurement instrument that tries to measure a characteristic or attitude that is believed to range across a continuum of values and cannot easily be directly measured [31]. It is often used in clinical research to measure the intensity or frequency of various symptoms [32]. VAS graded by the patient from 0 to 10, where 0 being no tinnitus, 1–3 (mild), 4–6 (moderate) and 7–10 (severe).

Transcutaneous electrical nerve stimulation (TENS) via preauricular skin (mainly concha) was conducted. The positive electrode placed ipsilateral to the tinnitus side. For patients with bilateral tinnitus, the positive electrode placed on the right side. TENS involved biphasic rectangular stimulation at 6 Hz for 10 min, immediately succeeded by 40 Hz stimulation for another 10 min. Pulses with a pulse width of 250 µs were applied for both actual stimulations. The TENS stimulation intensity is gradually increased until the patient clearly experiences paresthesia, at which point it is lowered to subthreshold. Number of sessions was two sessions per week. Some patients received only one session and others received up to eleven sessions.

### Statistical analysis

Data entry and data analysis were done using SPSS version 22 (Statistical Package for Social Science). Data were presented as number, percentage, mean, standard deviation, median, range and IQR (Interquartile Range). Mann-Whitney test was used to compare quantitative variables between two groups and Kruskal Wallis test for more than two groups. Wilcoxon Signed Ranks test was used to compare quantitative variables between before and after TENS. Spearman correlation was done to measure correlation between quantitative variables. *P*-value considered statistically significant when *P* < 0.05.

## Results

A total of 64 subjects suffering from subjective tinnitus were included in this study, 38 (59.4%) male and 26 (40.6%) female. The age ranged between 20 and 60 years. Fifteen subjects out of the study group suffering from chronic illness (diabetes mellitus, hypertension or both) (Table 1).

**Table 1:** Demographic data of the study group

Personal data	No. (64)	%
**Age: (years)**		
< 40	41	64.1%
≥ 40	23	35.9%
Mean ± SD	35.11 ± 13.12	
**Gender**:		
Male	38	59.4%
Female	26	40.6%
**Chronic diseases**:		
Yes	15	23.4%
No	49	76.6%

**Fig. 1:**
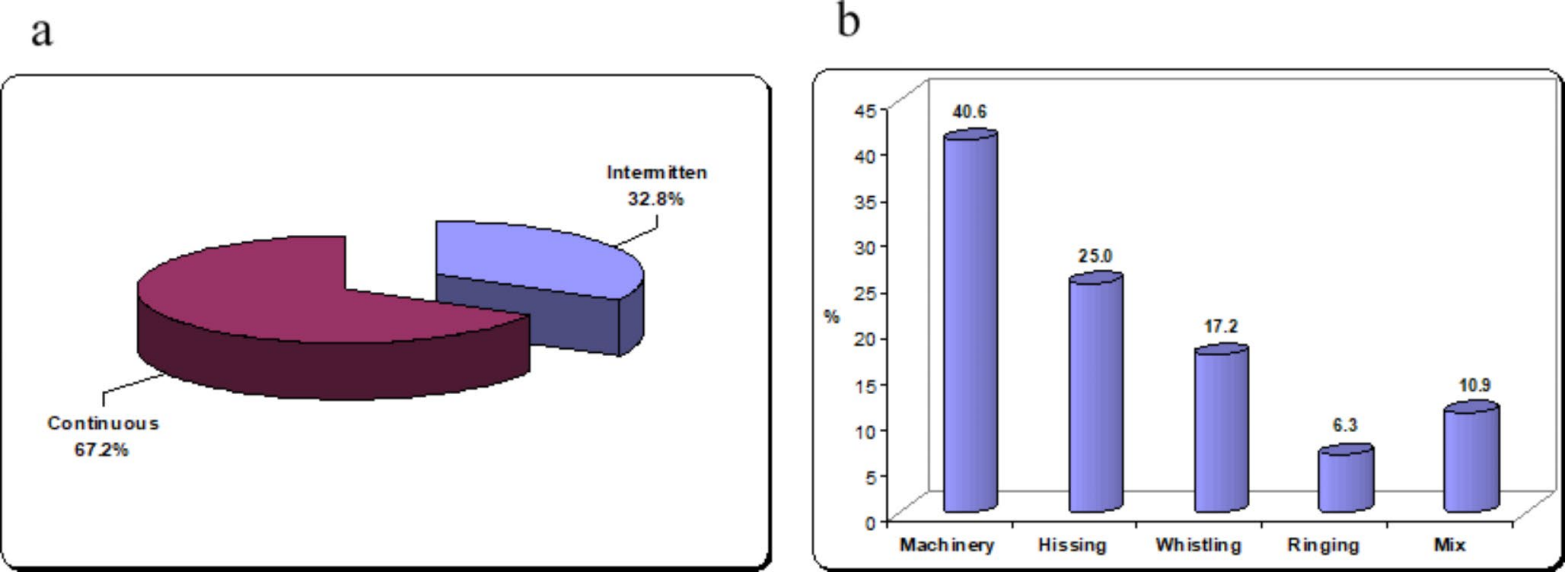
Character of tinnitus in the study group. **a**: continuous versus intermittent tinnitus. **b**: distribution of tinnitus sound

The characteristics of tinnitus in the study group are shown in Table 2. It was noticed that the most of the subjects (67.2%) have continuous tinnitus in comparison to intermittent type, and the machinery tinnitus represent 40.6% (Fig. 1). In thirty-three subjects, there is associated headache with tinnitus.

**Table 2:** Tinnitus characteristics of the study group

Tinnitus characteristics	No. (64)	%
**Tinnitus type:**		
Intermittent	21	32.8%
Continuous	43	67.2%
**Laterality**:		
Right	12	18.8%
Left	26	40.6%
Bilateral	26	40.6%
**Duration of tinnitus: (months)**		
< 6	15	23.4%
6–12	18	28.1%
13–24	13	20.3%
> 24	18	28.1%
**Tinnitus sound**:		
Machinery	26	40.6%
Hissing	16	25.0%
Whistling	11	17.2%
Ringing	4	6.3%
Mix	7	10.9%
**Associated symptoms**:≠		
Headache	33	51.6%
Dizziness	19	29.7%
Ear pressure	9	14.1%
No symptoms	12	18.8%

≠ more than one associated symptom

The severity of tinnitus using VAS before TENS was mild, moderate and severe in seven, 33, and 24 patients, respectively. Comparing the result of VAS before and after TENS showed that 45 out of 64 (70.31%) patients had improvement, from them nine patients had a complete reduction of tinnitus. Eighteen patients had no improvement and one patient felt worsening of tinnitus but returned to the initial level later. Wilcoxon Signed Rank test show a statistically significant difference in tinnitus loudness before and after TENS as assessed by VAS (Table 3).

**Table 3:** Visual analogue scale in before TENS, after TENS and the difference between them

	VAS			*P*-value
	Before TENS	After TENS	Difference	
**Median (Range)**	6.0 (2.0–9.0)	3.0 (0.0–9.0)	3.0 (-2.0-8.0)	0.000*

To study the effect of different tinnitus related variables; personal variables like (age, gender, and presence of chronic disease) and tinnitus characteristics (type, localization, duration, sound, associated symptoms) on the degree of tinnitus loudness before and after TENS stimulation (tinnitus reduction), Mann-Whitney and Kruskal Wallis test were used (Tables 4 and 5).

**Table 4:** Relation between personal data and VAS difference

Personal data	VAS difference	*P*-value
	Median (IQR)	
**Age: (years)**		
< 40	3.0 (2.0–5.0)	0.322
≥ 40	3.0 (0.0–5.0)	
**Gender**:		
Male	3.0 (0.0–5.0)	0.829
Female	3.0 (2.0–5.0)	
**Chronic diseases**:		
Yes	3.0 (0.0–5.0)	0.572
No	3.0 (0.0–5.0)	

**Table 5:** Relation between tinnitus characteristics and VAS difference

Tinnitus characteristics	VAS difference	*P*-value
	Median (IQR)	
**Tinnitus type:**		
Intermittent	3.0 (0.0–5.0)	0.782
Continuous	3.0 (0.0–5.0)	
**Laterality**:		
Right	3.5 (2.5-5.0)	
Left	3.0 (0.0–5.0)	0.932
Bilateral	3.0 (0.0–5.0)	
**Duration of tinnitus: (months)**		
< 6	3.0 (0.0–4.0)	
6–12	4.0 (2.0–5.0)	0.440
13–24	2.0 (0.0–5.0)	
> 24	4.0 (0.0–5.0)	
**Character of tinnitus**:		
Machinery	3.0 (0.0–5.0)	
Hissing	3.0 (0.0–6.0)	
Whistling	5.0 (3.0–7.0)	0.316
Ringing	2.5 (1.0–3.0)	
Mix	2.0 (0.0–5.0)	
**Associated symptoms**:		
Yes	3.0 (0.0–5.0)	0.909
No	3.5 (1.0–5.0)	

Regarding the personal variables, it was found that there is no statistically significant relation between the studied variables and the degree of tinnitus reduction (Table 4). Regarding the tinnitus characteristics, it was found that the degree of tinnitus reduction (median) is higher when tinnitus is presented in right ear in comparison to left or bilateral. Also, it was found that the median of tinnitus reduction was highest when the tinnitus is whistling in character followed by machinery and hissing, then ringing and the lowest reduction was found when tinnitus is a mix of more than one of them, however these differences are not significant (Table 5).

## Discussion

Tinnitus is a disease with significant heterogeneity in terms of etiology, perception, and degree of severity, ranging from mild annoyance to terrible impact on daily life [33]. To our knowledge and up to date, there are no universally known therapies (medical or non- medical therapies) with precise therapeutic effects for tinnitus, thereby making tinnitus still a challenging disease to be treated [5, 13, 14]. Therefore, the generation of robust evidence regarding the efficacy and safety of ta-VNS on alleviation of tinnitus will guide clinicians, to determine whether ta-VNS can be an optional treatment approach for tinnitus, thereby enriching the current treatment strategies.

In this study 64 subjects suffering from tinnitus with normal hearing were subjected to TENS of the auricular branch of vagus nerve. The severity of tinnitus before and after TENS was assessed by VAS. Our results showed a statistically significant reduction in severity of tinnitus after TENS. 45 out of 64 (70.31%) patients had improvement, from them nine patients had a complete reduction of tinnitus. Stimulation of the vagus nerve modulates the release of norepinephrine and acetylcholine [34, 35]. These neuromodulators enhance neuroplasticity by modulating the cortex, hippocampus, and amygdala. It has also been reported that norepinephrine and acetylcholine can affect the selective plasticity of auditory cortical neurons [36–38].

According to the study of Yang et a l [39], they revealed that the threshold of auditory brainstem response could be modulated by ta-VNS combined with sound masking, and it is attributed to modulation of neurotransmitters such as gamma- aminobutyric acid, 5- hydroxytryptamine and acetylcholine in the inferior colliculus.

In last years, several studies have been conducted to explore the effect of ta-VNS (alone or adjunctively) on tinnitus. For example, some studies reported that ta-VNS combined with sound masking can significantly improve the handicap and psychological symptoms of tinnitus [27, 40], while other studies revealed that there was no clinically relevant improvement of tinnitus symptoms in patients with chronic tinnitus who received ta-VNS treatment [26, 41]. Various parameters of ta-VNS stimuli, such as different stimulation frequencies, pulses and stimulation duration as well as electrode placement may contribute in this differences between our study and others. Also, tinnitus heterogeneity and patient expectation have a role in this difference.

The prediction of tinnitus therapy outcome is difficult. To our knowledge, several studies addressing different therapies and using different variables reported significant predictors but there is no study aiming to assess tinnitus related variables effect on ta-VNS treatment. The second aim in our study was to evaluate the effect of different tinnitus related variables on the degree of alleviation of tinnitus.

Our results revealed that the amount of tinnitus reduction is not related to the personal variables (age, gender, and presence of chronic disease) so age and gender could not be used as predictors for TENS results. On the other hand, some of studied tinnitus characteristics (localization and sound) has a difference effect on the amount of tinnitus reduction. It was found that the degree of tinnitus reduction is higher when tinnitus is presented in right ear in comparison to left or bilateral. Also, the tinnitus reduction was highest when the tinnitus is whistling in character followed by machinery and hissing, then ringing and the lowest reduction was found when tinnitus is a mix of more than one of them but these differences are not significant.

As we mentioned before, some studies revealed the effect of different variables on the outcome of different tinnitus therapy. Regarding personal variables, age was a significant predictor in some studies, but with only partly corresponding results [42]. Caffier et al., described a U-shaped relationship of age and outcome, they found that younger and older patients displayed a highly significant beneficial response to therapy that was not seen in middle-aged persons [43]. In the study by Graul et al., responders to treatment were younger [44]. Also, gender turned out to be a predictor in several studies, but also with inconsistent results [42]. Treatment outcome was reported to be better in men [45], but also women [46]. In another study, the gender effect on treatment depended on the type of therapy [47]. Moreover, it was found that being female in combination with long tinnitus duration predicted less benefit of treatment [48].

Regarding the tinnitus characteristics, some studies found that a shorter duration of tinnitus was a beneficial predictor [43, 49]. In one study using TENS of C2, Vanneste et al., found that the amounts of transient tinnitus suppression is independent of tinnitus type (pure tone or narrow band noise), tinnitus side (unilateral or bilateral), and tinnitus duration as well as gender, which makes it difficult to decide who might benefit from TENS and who not [50].

By the end of the discussion, we could conclude that ta-VNS is an effective treatment of subjective tinnitus in 70.31% of the studied group from them 20% have a complete reduction of tinnitus, but we could not assess for how long this residual inhibition persists due to lack of long term follow up. Residual inhibition means the suppression of tinnitus loudness for a period. However, it is difficult to decide who might benefit from ta-VNS, patients with unilateral tinnitus on right ear and those with whistling sound have more reduction in tinnitus loudness than others. TENS represents a safe and feasible treatment option for tinnitus and might be commendable among the spectrum of interventions developed for tinnitus.

## Limitation

our study has several potential limitations. First, lack of longer follow up to assess the duration of residual inhibition (long term effect). Second, there is no placebo-controlled group; hence, the significance of the effects can only be investigated through before-and-after comparison. Third, the outcome measures are self-assessments instead of objective measures. To some extent, objective measures carry out relatively convincing evidence. However, visual analogue scale [56], has been much used in most of the studies. The VAS is believed to range across a continuum of values and cannot easily be directly measured [31]. It is often used in clinical research to measure the intensity of various symptoms [32]. Fourth, it is a non-blind trial; so the psychological factors of the clinician and subjects can cause bias. Finally, this study is a single center study conducted on a small sample so it does not lend itself to great generalizability.

Additional studies will be necessary to confirm and utilize the results of the study. A large sample, multicenter, a comprehensive and standardized assessment of patients and objective measures to assess the effectiveness of treatment and long term follow up should be included in a future study.

## Data Availability

Data generated or analysed during this study are included in this published article and available from the corresponding author on reasonable request.

## Abbreviations

ABVN: Auricular branch of the vagus nerve
ta-VNS: Transcutaneous auricular vagus nerve stimulation
TENS: Transcutaneous electrical nerve stimulation
VAS: Visual analogue scale
